# P-186. Trends in Antimicrobial Resistance Among Adults Across 6 Hospitals in Argentina, Brazil, and Chile, 2018-2022

**DOI:** 10.1093/ofid/ofae631.390

**Published:** 2025-01-29

**Authors:** Charlene R Siza, Garrett W Mahon, Twisha S Patel, Maria I Garzon, Icaro Boszczowski, José M Munita, Fernanda C Lessa, Matias Chiarastelli Salomao, Giovanna Marssola, Bruno Tavares, Daniela Verónica Hernandez, Anne S Peters, Maria Spencer, Olivia L McGovern

**Affiliations:** Centers for Disease Control and Prevention, Atlanta, Georgia; Centers for Disease Control and Prevention, Atlanta, Georgia; Centers for Disease Control and Prevention, Atlanta, Georgia; Hospital Privado Universitario de Córdoba, Cordoba, Cordoba, Argentina; Hospital Alemão Oswaldo Cruz, São Paulo, Sao Paulo, Brazil; Clínica Alemana - Universidad del Desarrollo, Santiago, Chile; CDC, Atlanta, Georgia; Hospital das Clínicas da Faculdade de Medicina da Universidade de São Paulo, Sao Paulo, Sao Paulo, Brazil; Hospital Alemão Oswaldo Cruz, São Paulo, Sao Paulo, Brazil; Hospital das Clínicas Faculdade de Medicina da USP and Hospital Alemão Oswaldo Cruz, São Paulo, Sao Paulo, Brazil; Hospital Privado Universitario de Córdoba, Cordoba, Cordoba, Argentina; Universidad del Desarrollo, Santiago, Region Metropolitana, Chile; Genomics & Resistant Microbes (GeRM), Instituto de Ciencias e Innovación en Medicina, Facultad de Medicina Clínica Alemana, Universidad del Desarrollo, Chile; Millennium Initiative for Collaborative Research on Bacterial Resistance (MICROB-R), Santiago, Region Metropolitana, Chile; U.S. CDC, Atlanta, Georgia

## Abstract

**Background:**

In 2019, an estimated 1.27 million deaths were attributed to bacterial antimicrobial resistance (AR) globally. During the Coronavirus Disease 2019 (COVID-19) Pandemic, additional strain was placed on healthcare systems that may have led to further increases in AR. We evaluated AR prevalence before and after the onset of COVID-19 in 6 healthcare facilities (HCF) in South America.

**Figure:**
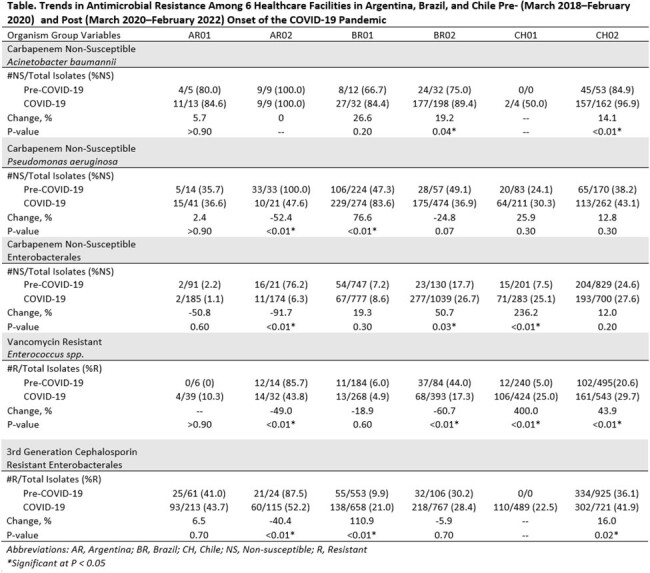

**Methods:**

We conducted an ecological evaluation of HCF-wide AR prevalence in 4 private and 2 public HCFs in Argentina, Brazil, and Chile; 2 HCFs per country. We calculated percentage of non-susceptible (NS) or resistant (R) to select antibiotics among clinical isolates of *Acinetobacter baumannii, Pseudomonas aeruginosa, Enterococcus* spp., and Enterobacterales cultured from adult acute care inpatients. We compared percentage-NS or -R between the periods pre- (March 2018–February 2020) and post- (March 2020–February 2022) onset of the COVID-19 pandemic. Chi-square or Fisher’s exact tests were used with statistical significance set at a two-tailed *p*< 0.05.

**Results:**

When comparing 2020-2022 to 2018-2020, 2/6 hospitals showed statistically significant increases (+14.1–19.2%) in carbapenem-NS *A. baumannii*. For carbapenem-NS *P. aeruginosa,* 1/6 hospitals showed a significant increase (+76.6%) and 1/6 showed a significant decrease (-52.4%). For carbapenem-NS Enterobacterales, 2/6 hospitals had significant increases (+50.7–236.2%) and 1 exhibited a significant decrease (-91.7%). For vancomycin-R *Enterococcus* spp., 2/6 hospitals had significant increases (+43.9–400.0%) and 2/6 hospitals had significant decreases (-49.0-60.7%). For Enterobacterales-R to 3^rd^ generation cephalosporins, 2/6 hospitals showed significant increases (+16.0–110.9%) and 1/6 hospitals had a significant decrease (-40.4%) (Table).

**Conclusion:**

Among the isolates from our study hospitals, we noted not only significant increases but also significant decreases across multiple AR profiles associated with multi-drug resistance. These data suggest that the pandemic may have led to changes in resistance among both gram-negative and gram-positive bacteria. Given the variability in these changes, further evaluation of infection rates would be valuable to confirm these findings.

**Disclosures:**

**José M Munita, MD**, MSD: Grant/Research Support|Pfizer: Grant/Research Support

